# Neutrophil/lymphocyte and platelet/lymphocyte ratio in seropositive women for human immunodeficiency virus (HIV) and human papillomavirus (HPV) coinfection

**DOI:** 10.1590/S1678-9946202466067

**Published:** 2024-12-06

**Authors:** Karina Donato Fook, Maria José Abigail Mendes Araújo, Alessandra Costa de Sales Muniz, Mônika Machado de Carvalho, Ana Cléa Cutrim Diniz de Morais, Deborah Rocha de Araújo, Sulayne Janayna Araújo Guimarães, Camila Penha Abreu Souza, Carla Déa Trindade Barbosa, Maria Fernanda Lima Bertolaccini, Ilka Kassandra Pereira Belfort, Fernanda Ferreira Lopes, Sally Cristina Moutinho Monteiro

**Affiliations:** 1 Universidade Federal do Maranhão, Programa de Pós-Graduação em Saúde do Adulto, São Luís, Maranhão, Brazil; 2 Universidade Federal do Maranhão, Hospital Universitário, Laboratório de Análises Clínicas e Histocompatibilidade, São Luís, Maranhão, Brazil; 3 Secretaria Municipal de Saúde de São Luís, São Luís, Maranhão, Brazil; 4 Faculdade Laboro, São Luís, Maranhão, Brazil

**Keywords:** Human immunodeficiency virus, Human papillomavirus, Oncotic cytology, Biomarkers

## Abstract

This study aims to investigate the possible association between neutrophil/lymphocyte and platelet/lymphocyte ratio in women with HIV, undergoing antiretroviral treatment, with HPV coinfection. This is a cross-sectional study with HIV positive women; their biological samples were collected for laboratory tests (complete blood count) and oncotic cytology for detection of HPV DNA, by PCR-Nested (PGMY and GP primers). Viral load and CD4 and CD8 T-cells counts were obtained from medical records. The data were analyzed, comparing the two groups: those with coinfection and those without it. From 82 HIV seropositive women, 50% exhibited HPV coinfection and 12.2% of coinfected patients had cervical cell alterations. Quantification of viral load, CD4 and CD8 T-cells count, CD4 / CD8 ratio and neutrophil/lymphocyte (NLR) and platelet/lymphocyte (PLR) ratio presented significant differences between groups (p<0.05). The predicting power of NLR and PLR in differentiating HIV/HPV coinfection which demonstrated differences between groups (AUC of 0.882 and 0.776 for NLR and PLR, respectively). There is a relation between the neutrophil/lymphocyte and platelet/lymphocyte ratio with HIV/HPV coinfection in women undergoing antiretroviral treatment, suggesting a state of greater and persistent systemic inflammation, reflecting as a biomarker for screening and monitoring these patients.

## INTRODUCTION

Human immunodeficiency virus (HIV) infection and acquired immunodeficiency syndrome (AIDS) are public health issues, with 37.9 million HIV-positive individuals in the world and 1.7 million new cases per year^1^. HIV positive women are more susceptible to opportunistic infections and neoplasms, such as human papillomavirus (HPV) infection, when compared to HIV-negative women^2-4^. HIV infection facilitates HPV infection and replication, making it more severe and persistent^5-7^. Furthermore, low CD4 T-cell count and high HIV viral load are independent risk factors for HPV. Such findings are consistent with an important role for the immune response and inflammatory markers in the control^8^ and prognostic evaluation of HPV infection.

HIV-positive people on antiretroviral treatment have a persistent degree of systemic inflammation and low activation of the immune system^9^. Elevated levels of inflammatory markers can predict adverse events, as well as low CD4 T-cells and plasma replication of HIV^10,11^.

Recently, two biomarkers of blood parameters (neutrophil/lymphocyte [NLR] ratio and platelet/lymphocyte [PLR] ratio) have been shown to be indicative of systemic inflammation, as well as predictive biomarkers of morbidity and mortality for cardiovascular (CVD) and non-cardiovascular diseases, such as cancer and chronic kidney disease (CKD)^12-14^. In a cohort of HIV-positive people, NLR and PLR were associated with risk of death in cases of solid tumors or lymphoma^15^, while NLR was considered an independent prognostic factor for cancer-free survival in patients with cervical intraepithelial neoplasia (CIN)^16^.

The relation between systemic inflammatory process and development of cancer is well established. Moreover, NLR and PLR are biomarkers of poor prognosis in different organic disorders, with such biomarkers also being related to a cell-mediated immune response in HPV infection control^12-14,16^. Therefore, this study aimed to investigate the possible association between NLR and PLR with the presence of the human papillomavirus in women with human immunodeficiency virus undergoing antiretroviral treatment.

## MATERIALS AND METHODS

### Ethical approval and consent to participate

This research was approved by the Research Ethics Committee of the University Hospital of the Federal University of Maranhao (Hospital Universitario da Universidade Federal do Maranhao - HU-UFMA, Sao Luis, Maranhao State, under Nº 2.776.970 and C.A.A.E Nº 70989617.4.0000.5086). Investigators explained the study objectives and methodology; all participants were adults and provided written informed consent to take part in the study. Consent forms were kept separately from questionnaires and biological samples to guarantee the ethical standards.

### Study population

This is a cross-sectional survey with 82 HIV-positive women. All participants were served at two Reference Centers for HIV treatment in the Sao Luis city, Maranhao State, Brazil. Inclusion criteria were a) age ≥18 years; b) more than five years since HIV diagnosis; c) be in treatment with antiretroviral therapy (ART) for the last three years and at the time of sample collection; d) no CD4 nadir <50 cells/mm^3^. Furthermore, pregnant women, women with contraindications for Pap smears (for example, current use of vaginal eggs, menstruation, vaginal douches in the last 24 hour and hysterectomies) were not included.

All participants answered a sociodemographic questionnaire (age, educational level, sexarche, marital status, number of sexual partners during lifetime and type of sexual practice, among others), current history of the disease, drug treatment, information about other sexually transmitted infections and habits (use of tobacco and / or illicit drugs, among others).

### Laboratorial investigation

#### Sample collection

Samples of cervical swabs were collected for cytological and molecular biology exams to detect human papillomavirus. Blood samples were subjected to performance evaluation of the complete blood count.

The CD4 and CD8 T-cells count (cells/mm^3^), with flow cytometry, and detectable viral load (copies/mL) data were obtained from electronic medical records of the hospital reference service. The CD4 and CD8 T-cells count and viral load results retrieved from patients’ electronic records were selected according to their temporal proximity to the time of testing for HPV on patients’ blood samples (conducted in up to three months).

#### Laboratory tests and oncotic cytology

The blood counts were processed by an Advia 2120 analyzer (Siemens Healthcare Diagnostics, Deerfield, IL). The neutrophil/lymphocyte ratio (NLR) was obtained by dividing the absolute neutrophil count by the absolute lymphocyte count. The platelet/lymphocyte ratio (PLR) was obtained by dividing the absolute platelet count by the absolute lymphocyte count. Oncotic cytology assays were performed using cytological smears obtained with Ayre spatula (ectocervical sample) and endocervical brush (endocervical sample), extended on a glass slide, fixed with ethanol and stained using the Pap smear. The 2001 Bethesda System was used for reporting cervical or vaginal cytologic diagnoses.

#### HPV DNA testing

DNA extraction was performed following the manufacturer’s guidelines (Biopur Mini Spin Plus Extraction Kit, Biometrix, PR, Brazil). The Nested Polymerase Chain Reaction (PCR) technique was performed using a GeneAmp PCR System 9700 thermocycler (Applied Biosystems, Thermo Scientific, California, USA), with PGMY 09 and 11 primers (amplifying 450bp sequences from the region L1 of viral DNA) and GP + 5 and GP + 6 (amplifying 190bp sequences of the L1 region of viral DNA). Samples known as HPV-positive were used as a positive control, whereas ultrapure water was the negative control.

For the amplification reaction using the PGMY09/11 primers, the initial denaturation took place in 2 min at 95 °C followed by 40 cycles of denaturation for 40 s at 95 °C, 40 s of annealing at 55 °C, 40 s of extension at 72 °C. The second round of the Nested PCR was carried out with the GP5 + / GP6 + primers with an initial denaturation at 95 °C for 4 min followed by 45 cycles of denaturation at 95 °C for 45 s, annealing at 40 °C for 1 min and extension at 72 °C for 1 min^17^. The amplification products were evaluated by electrophoresis on 1.5% agarose gel in TBE 1X buffer for 30 minutes at 5 V / cm in a horizontal vat (Life Technologies, Carlsbad, CA, USA). The bands were stained with 0.1% Red Gel (Invitrogen) and visualized using an ultraviolet transilluminator (BioRad Laboratories, Hercules, CA, USA).

## Data analysis

Data were analyzed with IBM SPSS^®^ Statistics program version 24.0 Windows (SPSS Inc., Chicago, IL) and level of significance was p<0.05 (two-tailed). The Chi-square test and Fisher’s exact test were used for group comparison for categorical variable, between HPV+ (positive) or HPV− (negative) participants. The normal distribution of numerical variables was verified, and, after that, Student’s *t*-test was used to verify the differences between the means of the categorical groups evaluated.

## RESULTS

The sample consisted of 82 HIV-positive women undergoing antiretroviral treatment, of which 41 (50%) were positive for DNA-HPV. Table 1 describes the socioeconomic status of HPV-positive and HPV-negative participants, with a total of 68.3% participants having studied for less than 9 years. In the group of HPV-positive women (HIV/HPV coinfection), it was found that 51.2% live with a partner; 70.7% have a family income of up to 1 minimum wage (Brazilian currency); 51.2% had their first sexual intercourse between 12 and 15 years old; 53.7% had up to three sexual partners during their lifetime; 48.8% use condoms during sexual intercourse; 51.8% practice oral/anal intercourse; 19.5% are smokers; 7.6% had more than four children and 43.9% had an abortion episode. There were no statistically significant differences in socioeconomic aspects and health data between the study groups (Table 1).

**Table 1:** Socioeconomic and health data of women with and without HIV/HPV coinfection, Sao Luis, Maranhao State, Brazil, 2019.

	HPV positive (%) N=41	HPV negative (%) N=41	p-value
**Age**			
18 to 34 years	29.3	22.0	0.691
35 to 49 years	39.0	39.0	
More than 50 years	31.7	39.0	
**Education Level**			
≤ 9 years	68.3	68.3	0.648
> 9 to 12 years	22.0	26.8	
> 12 years	9.8	4.9	
**Marital Status**			
With partner	51.2	43.9	0.507
Without partner	48.8	56.1	
Family income *			
Up to a minimum wage	70.7	65.9	0.853
1 to 3 minimum wages	24.4	26.8	
3.1 to 5 minimum wages	4.9	7.3	
**Age of first sexual intercourse**			
< 12 years	0	0	0.825
12 to 15 years	51.2	48.8	
> 15 years	48.8	51.2	
**Number of sexual partners**			
≤ 3	53.7	46.3	0.775
4 to 6	29.3	31.7	
≥ 7	17.1	22.0	
**Condom use**			
Yes	48.8	48.8	1.0
No	51.2	51.2	
**Anal/Oral intercourse**			
Yes	53.7	39.0	0.184
No	46.6	61.0	
**Contraceptive use**			
Yes	5.1	2.5	0.541
No	94.9	97.5	
**Smoking**			
Yes	19.5	7.3	0.125
No	80.5	92.7	
**Parity**			
Nulliparity	4.9	14.6	0.258
1 to 3	19.5	12.2	
≥4	75.6	73.2	
**Abortion**			
Yes	43.9	41.5	0.823
No	56.1	58.5	

Data presented in proportion. Tests used: Chi-square and Fisher’s exact test. *Minimum wage in Brazil, 2019 = $ 998.00.

**Figure 1:**
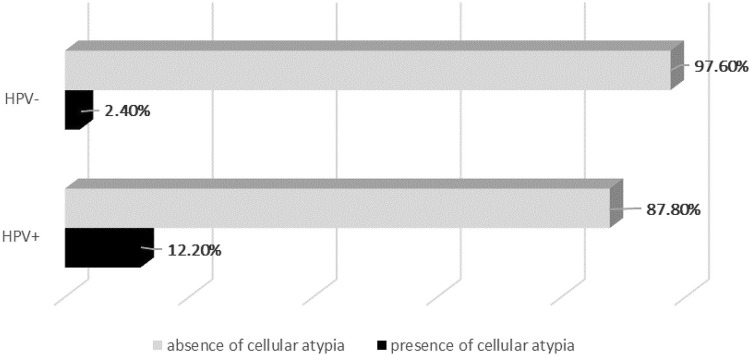
Frequency of atypical cells (%) detected in oncotic cytology assays of cervical smear in women with HIV. Sao Luis, Maranhao State, Brazil, 2019.

Oncotic cytology assays indicated atypical cells in 12.2% of HPV-positive participants and 2.4% of HPV-negative participants (Figure 1), with statistical difference between the groups (Fisher’s exact test, p = 0.010). In the HPV-positive participants, of those Pap smear showing some atypical squamous cells of uncertain significance (ASC-US), 4.9% presented high-grade squamous intra-epithelial lesion (HSIL) and 2.4% presented low grade squamous intraepithelial neoplasia (LSIL). Among HPV-negative participants, a total of 2.4% Pap smear results presented ASC-US.

Although higher values were observed in the group with HIV/HPV coinfection, no statistically significant difference was found for the following variables: total leukocytes; total neutrophils; total platelets; hemoglobin concentration; and hematocrit between HPV-positive and HPV-negative participants. When CD4 T-cell, CD8 T-cell and the CD4/CD8 ratios were evaluated, there were statistically significant differences (p = 0.02; p = 0.02 and p = 0.03 respectively). The elevation neutrophil/lymphocyte ratio (p = 0.04), platelet/lymphocyte ratio (p = 0.01) and viral load (p = 0.04) also showed a statistical difference between groups (Table 2). All these parameters show higher values among coinfected participants.

**Table 2:** Laboratorial data of women with and without HIV/HPV coinfection. Sao Luis, Maranhao State, Brazil, 2019.

	HPV-positive N= 41	HPV-negative N=41	*p*-value
**CD4 T-cell**	581.66±270.27	767.51±251.97	**0.02**
**CD8 T-cell**	945.24±424.24	744.35±326.84	**0.02**
**CD4/CD8 ratio**	0.81±0.52	1.07±0.60	**0.03**
**Leucocytes**	5982.78±3453.58	5725.57±1836.14	0.69
**Neutrophils**	3353.97±3274.31	3048.57±1417.21	0.61
**Lymphocytes**	2225.42±688.82	1863.58±568.97	**0.03**
**Hemoglobin**	12.88±1.09	12.72 ±1.60	0.62
**Hematocrit**	40.10±3.61	39.93±4.11	0.82
**Platelets**	329666.8 ±538276.6	260029.4±80268.29	0.44
**Neutrophil/lymphocyte ratio**	3.17±4.60	1.26±0.46	**0.04**
**Platelet/lymphocyte ratio**	154.34±94.24	111.11±34.23	**0.01**
**Viral load**	240.22±628.40	44.25±12.60	**0.04**

Data presented in mean ± Standard Deviation. Test used: Student’s t-test. CD4 = Cluster of differentiation 4; CD8 = Cluster of differentiation 8.

**Figure 2:**
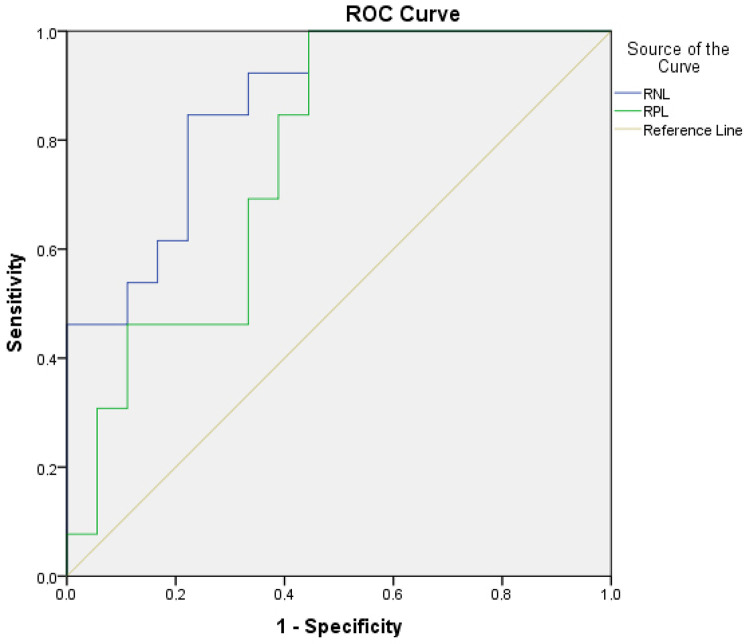
Receiver-operating characteristic curve for significant markers in the prediction of HPV presence in HIV-infected women. Sao Luis, Maranhao State, Brazil, 2019. NLR = Neutrophil lymphocyte ratio; PLR = Platelet lymphocyte ratio; ROC = Receiver-operating characteristic.

Receiver-operating characteristic curve models were assembled to evaluate the area under the curve (AUC). The predicting power of NLR and PLR in differentiating HIV/HPV coinfection were analyzed, which demonstrated differences between groups. The NLR presented an AUC of 0.882 (Sensitivity = 54.2%, Specificity = 100%, Cut off >2.014 and p>0.05) and the PLR AUC of 0.776 (Sensitivity = 90.5%, Specificity = 54.5, Cut off >107.06 and p>0.05), demonstrating that they are possible predictors of HPV coinfection (Figure 2).

## DISCUSSION

In this study, CD4 T- and CD8 T-lymphocyte count, CD4/CD8 ratio, viral load, lymphocyte count, the neutrophil/lymphocyte and platelet/lymphocyte ratios in HIV-positive women were associated with HPV coinfection. Inflammation can largely influence several stages of tumorigenesis, from tumor initiation to promotion and metastatic progression. Moreover, inflammatory cells and their mediators are in fact an essential component of the tumors microenvironment (TME), with cancer cells being able to induce inflammatory reactions through various mechanism^18^. HIV infection increases the risk of coinfections, such as HPV^5-7^, although some studies show no impact on HPV-associated disease^19,20^. Thus, it is still necessary to study HPV infection and its connection to HIV due to its great importance for public health.

This study has also found that atypical cells on oncotic cytology were more frequent in HPV-positive participants. The data obtained from the analyzed population showed a low proportion of coinfected participants (HIV/HPV) with cytological changes, however, it was more frequent in HIV/HPV coinfection participants, corroborating with literature data that detected a higher prevalence of cytological lesions in the population with HIV/HPV coinfection^21^. There were atypical cells on oncotic cytology in 12.2% of HPV-positive participants (higher proportion of ASCUS and HSIL). Hawes *et al*.^22^ found similar results in HIV-seropositive Africans with a rate of 12.3% of LSIL, while Zhang *et al*.^23^ found 16% with cellular changes.

Association between the risk of HPV infection and its persistence was observed in HIV-positive women, and cytological changes and progression to intraepithelial neoplasia are facilitated in HIV/HPV coinfection, so immunodeficiency is a predictor of cervical injury in this population^24^.

Lower CD4 T-cell count in HIV-positive women leads to a greater chance of HPV infection and evolution of cervical injury^25^. The data presented here demonstrated that coinfected participants had lower CD4 T-cell and CD4/CD8 ratios than participants without coinfection, while there was an increase in CD8 T-cell. Notably, due to antiretroviral treatment, the former were not more severely immunodeficient (<200 cells/mL), stressing the importance of medication adherence in this population^26^.

The NLR and PLR and viral load showed statistically significant values in HIV/HPV coinfection, even in the presence of antiretroviral treatment and the ROC curve models demonstrating that they are possible predictors for presence of the HPV. There was a reduction in CD4 T-lymphocytes, which in turn increased the number of neutrophils, CD8 T-lymphocytes and viral load, suggesting a state of greater and persistent systemic inflammation in individuals living with HIV/HPV coinfection. NLR and PLR ratios are emerging as new biomarkers of systemic inflammatory response used as noninvasive and low-cost prognostic indicators for solid tumors^18,27^. Furthermore, it is well established in the literature that the inflammatory state has an essential role in carcinogenesis^28-30^. However, few studies addressed this correlation in HIV-seropositive population with HPV coinfection without cancer.

NLR has been an important marker of systemic inflammation with predictive and prognostic value in several types of cancers^28^ and is an independent marker for survival of patients after tumor resection surgery in women with cervical intraepithelial neoplasia^16^. Cervical intraepithelial neoplasia is one of the most common neoplasms, of which HPV is the main causative agent, and the impaired neutrophil migration could be an early event in tumor development^31^. Compared with CIN and early-stage cervical cancer, leukocytosis, neutrophilia, lymphopenia, and NLR ≥ 5 were more frequently observed in advanced stage cervical cancer patients^32^.

In this study the results show an increase in NLR in HIV/HPV coinfected women reflecting a systemic inflammation with neutrophilia and lymphopenia. Thus, do our results reflect the contribution of NLR as a biomarker for screening an inflammation and monitoring cellular changes in these patients? This hypothesis needs to be further investigated.

Considering that platelets play a role in inflammation, function as effectors of injury in a variety of pulmonary disorders and syndromes, facilitate tissue repair and act in the growth and development of metastases of various cancers, their immunomodulatory properties have been investigated by many studies^33,34^. Bilir *et al*.^35^ found that higher PLR values were significantly correlated with decreased overall survival for patients with cervical cancer group and in the persistent human papilloma virus groups. Palaia *et al.* ^36^, by treating locally advanced cervical cancer, found that patients with low NLR and PLR showed significantly better responses to concomitant chemoradiation or neoadjuvant chemotherapy (NACT).

During HIV infection, an exaggerated systemic inflammatory response guides platelet dysfunction in which platelets are inappropriately activated, followed by immunological destruction and thrombocytopenia^37^. Platelets derived from HIV-infected individuals under stable antiretroviral therapy show increased mitochondrial dysfunction, activation of the intrinsic pathway of apoptosis and undermined granule secretion in response to thrombin^38^.

Two studies^37,38^ show that inflammation leads to an imbalance in the maintenance of platelet homeostasis, which supports the use of this hematological component as a possible alternative inflammatory biomarker. Therefore, this imbalance can explain why elevated PLR was associated with HIV/HPV coinfection in women in our study.

In clinical practice, NLR and PLR can estimate organic changes and the inflammatory response, and they can be considered recent biomarkers, which are associated with inflammation and aggregation pathways. However, more studies should be performed to confirm our outcomes. This study holds some limitations, namely: sample size and the lack of determination of serum concentrations of classic inflammatory markers such as interleukins (eg IL6) and ultra-sensitive C-reactive protein (CRPus). However, it is a pioneering study on the neutrophil/lymphocyte ratio and the platelet/lymphocyte ratio in patients living with HIV with and without HPV infection. Additionally, it is a preliminary study, and it could be considered a prototype for future studies of a prospective and randomized design.

## CONCLUSION

In women with HIV/HPV coinfection and under antiretroviral treatment, there is association between the neutrophil/lymphocyte and platelet/lymphocyte ratio suggesting a state of greater and persistent systemic inflammation and lower lymphocyte proportions; however, prospective randomized studies should be performed to confirm this hypothesis and to determine the usefulness of NLR and PLR in surrogacy for alternative inflammatory biomarkers.
